# LSD-YOLOv5: A Steel Strip Surface Defect Detection Algorithm Based on Lightweight Network and Enhanced Feature Fusion Mode

**DOI:** 10.3390/s23146558

**Published:** 2023-07-20

**Authors:** Huan Zhao, Fang Wan, Guangbo Lei, Ying Xiong, Li Xu, Chengzhi Xu, Wen Zhou

**Affiliations:** School of Computer Science, Hubei University of Technology, Wuhan 430068, China

**Keywords:** surface defect detection, YOLOv5s, Stem block, MobileNetV2 bottleneck, multi-scale feature fusion

## Abstract

In the field of metallurgy, the timely and accurate detection of surface defects on metallic materials is a crucial quality control task. However, current defect detection approaches face challenges with large model parameters and low detection rates. To address these issues, this paper proposes a lightweight recognition model for surface damage on steel strips, named LSD-YOLOv5. First, we design a shallow feature enhancement module to replace the first Conv structure in the backbone network. Second, the Coordinate Attention mechanism is introduced into the MobileNetV2 bottleneck structure to maintain the lightweight nature of the model. Then, we propose a smaller bidirectional feature pyramid network (BiFPN-S) and combine it with Concat operation for efficient bidirectional cross-scale connectivity and weighted feature fusion. Finally, the Soft-DIoU-NMS algorithm is employed to enhance the recognition efficiency in scenarios where targets overlap. Compared with the original YOLOv5s, the LSD-YOLOv5 model achieves a reduction of 61.5% in model parameters and a 28.7% improvement in detection speed, while improving recognition accuracy by 2.4%. This demonstrates that the model achieves an optimal balance between detection accuracy and speed, while maintaining a lightweight structure.

## 1. Introduction

Steel strips are a crucial product in the steel industry and serve as a foundational material in areas such as bridge engineering, shipbuilding, and automobile manufacturing [1]. The quality of steel strips directly impacts the performance and lifespan of various infrastructure systems. However, during production and transportation, the surface of steel strips may present multiple defects. These defects not only compromise the quality of the steel strips, but also contribute to inaccuracies in subsequent processing steps [2,3]. The timely and accurate identification of defects is an effective way to improve the quality and efficiency of steel strip production. Therefore, defect detection in industrial production carries significant practical value. Steel strip surface damage types are complex and diverse, and these defects may not be readily discernible [4], thereby posing certain challenges to detection.

The techniques employed for identifying surface damage on steel strips can be categorized into three groups, manual detection, automated detection, and artificial-intelligence-based detection. Initially, defect detection was mainly accomplished through manual labor, which necessitated prolonged periods of high-intensity labor for workers on the production line. This not only resulted in inefficiencies, but also increased the likelihood of wrong and missed detections [5]. As a result, the quality of the steel strips could not be well-guaranteed. With the development of automatic detection technology, eddy current testing, infrared thermography, magnetic flux leakage testing, ultrasonic testing, etc., have become commonly used in industrial production. Ghanei et al. [6] utilized eddy current testing to determine the martensite percentage of dual-phase steels and evaluate their mechanical properties. Keo et al. [7] employed a microwave excitation system coupled with infrared thermography for detecting vertical reinforcements in concrete. Zhang et al. [8] utilized an open-ended rectangular waveguide based on microwave non-destructive testing for detecting defects in thick-coated steel plates. However, these methods still have the limitations of material and the inability to accurately classify defects, making it difficult to recognize defects accurately and efficiently.

In recent years, artificial intelligence techniques have been widely used in defect detection. Current methods for detecting damage can be generally classified into two main categories: traditional machine-learning-based object detection [9] and deep-learning-based object detection [10]. For instance, Hussain et al. [11] proposed an object recognition model based on intelligent deep learning and an improved whale optimization algorithm (WOA). In their study, a data augmentation approach was first employed to address the imbalance in object classes. Then, the DenseNet201 network was enhanced, and an improved WOA was proposed to select the best features. The application of these methodologies has led to a substantial enhancement in the accuracy of the model. The traditional methods extract the damage features manually [12,13], such as Local Binary Pattern (LBP), Histogram Oriented Gradient (HOG), Grey-level Co-occurrence matrix (GLCM), etc., followed by the classification, which improves the efficiency of damage identification to some extent. Gola et al. [14] extracted textural features and morphological parameters from steel structures, utilizing a support vector machine algorithm to classify the microstructure of low-carbon steels. Luo et al. [15] proposed a new generalized completed local binary model framework for feature extraction. Furthermore, Ashour et al. [16] extracted multidirectional shearlet features from hot-rolled steel strip images, followed by Gray-level Co-occurrence Matrix (GLCM) calculations. However, these methods are susceptible to external environmental influences, such as lighting conditions and backgrounds. Moreover, the accuracy of recognition heavily depends on feature engineering, resulting in poor robustness and model generalization.

Compared with traditional methods, deep learning detection methods can automatically learn features from raw data with higher accuracy and efficiency [17,18], and they exhibit increased resistance to external interference. Deep learning detection algorithms can be broadly categorized into two main categories. One is a two-stage algorithm based on candidate regions. For example, Zhou et al. [19] improved the Fast R-CNN model’s ability to detect surface diseases on steel strips, combining a novel residual atrous spatial pyramid pooling module with the feature pyramid network to enhance multiscale feature fusion. Akhyar et al. [20] introduced deformable convolution, deformable RoI pooling, and guided anchoring RPN to optimize the cascade R-CNN algorithm. Selamet et al. [21] proposed a metal surface defect detection model that combines the Faster R-CNN algorithm with the shape from the shading method. The other is a regression-based single-stage algorithm. For instance, Guo et al. [22] introduced the TRANS structure into the backbone network and detection head of YOLOv5, aiming to combine features and global information effectively. Zhang et al. [23] detected and classified damaged hot-rolled steel strips using a network model based on YOLOv5s. Li et al. [24] improved the performance of the YOLOv5 algorithm by incorporating dense multiscale weighted feature fusion and ASPF modules. The above studies illustrate the significant advancements in deep learning detection algorithms and their broad applicability in the industrial production of steel strips.

Surface defect detection on steel strips is a critical application of object detection. However, current research primarily concentrates on enhancing the precision of surface defect classification [25,26], while simultaneously considering the recognition speed and lightweight design of the model can be challenging. Liu et al. [27] developed an end-to-end multiscale contextual detection model for identifying steel strip damage with multiple scales and complex backgrounds. Although this model can achieve real-time detection of steel strip damage, its effectiveness in detecting defects with irregular shapes and unclear boundaries is limited. Li et al. [28] proposed a surface defect detection approach based on YOLOv4. However, the detection model is complex and not ideal for implementation in devices with limited resources. Tian et al. [29] utilized key point estimation to determine the defect centers, which optimized the detection speed of the model. Nonetheless, the model’s performance is suboptimal when detecting ambiguous defects. Zhou et al. [30] proposed a lightweight detection mode, which performs well in terms of real-time and lightweight. However, this structure is prone to wrong and missed detections when detecting steel strip images with overlapping targets. Liu et al. [31] designed a TruingDet algorithm based on Fast R-CNN. They strengthened the ability of the detection model to identify and localize damage, but failed to address the issue of balancing classification and regression effectively.

Although the current methods for object detection have achieved some success, they still face the following challenges: (1) The speed of classification is limited by the complex network structure and large computational volume, leading to high latency. Furthermore, powerful computer hardware is required for model training, making it challenging to deploy on devices with limited memory and computation resources. (2) The detection accuracy is generally low for poorly characterized defects and small-scale targets. There are instances of missed detections for defects that are closely spaced and mutually occluded. To address the above challenges, we designed a practical defect detection method with fewer parameters to achieve a good balance between detection accuracy and speed. YOLOv5 is an open-source object detection model that addresses the issue of recognition and detection in industrial scenarios, offering both speed and compactness. This study aims to enhance the YOLOv5 model by reducing its complexity, boosting detection speed, and simultaneously improving accuracy. In this work, the major contributions are as follows:

- We developed a lightweight steel strip surface defect detection model, LSD-YOLOv5.
- We proposed a new, efficient feature extraction network by integrating the R-Stem module and CA-MbV2 module into the backbone network. This has led to a significant reduction in model parameters, while also improving the speed of detection.
- A smaller bidirectional feature pyramid network (BiFPN-S) was implemented in the model to effectively integrate feature information at multiple scales.
- We improved the recognition efficiency of overlapping targets by employing the Soft-DIoU-NMS prediction frame screening algorithm.

The rest of this paper is organized as follows. Section 2 details the proposed model LSD-YOLOv5 for steel strip surface defect detection. Section 3 evaluates the experimental results and compares our proposed model with state-of-the-art methods. Section 4 concludes this paper and describes the directions for future work.

## 2. Materials and Methods

### 2.1. Overall Framework of Steel Strip Defect Detection

The timely and efficient identification of surface defects is essential for improving the quality of steel strip production. However, current detection methods are limited in their ability to balance speed and accuracy while maintaining a lightweight model. To solve these issues, we proposed a lightweight network model, LSD-YOLOv5, for detecting steel strip surface damage. First, we found that using a simple convolution operation in the front end of the backbone network to extract the original image features may result in the loss of significant surface defect feature information. To enhance feature extraction, we proposed a shallow feature enhancement module called R-Stem. Second, the original network model exhibits a high parameter count and slow detection speed. Therefore, we designed a new CA-MbV2 structure that integrates the Coordinate Attention mechanism [32] into the lightweight MobileNetV2 bottleneck to achieve real-time detection while maintaining a lightweight structure. However, we found that the model exhibits low detection accuracy and high false detection rates for small-scale targets in practical strip defect identification. Our analysis was that the feature pyramid in the original model using the PANet structure [33] failed to fuse the features effectively. Consequently, we proposed a smaller bidirectional feature pyramid network (BiFPN-S), enabling more effective multi-scale feature fusion. Finally, to enhance the identification of overlapping targets, we used Soft-DIoU-NMS as a prediction frame screening algorithm.

### 2.2. Dataset Processing

**Figure 1:**
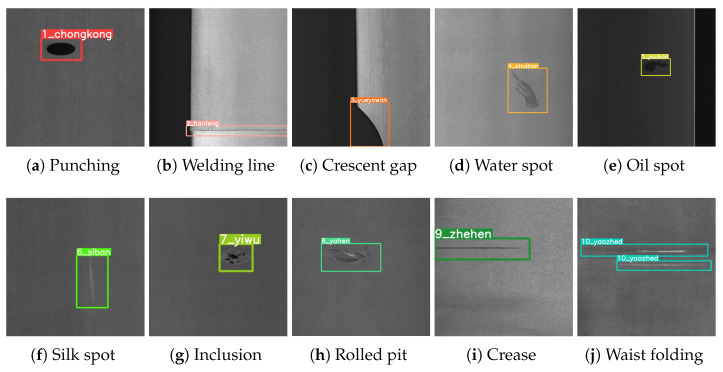
An example of the GC10-DET steel strip surface defect dataset.

In this study, we utilize the open-source GC10-DET dataset [34], which is accessible on GitHub (Website online: https://github.com/lvxiaoming2019/GC10-DET-Metallic-Surface-Defect-Datasets (accessed on 24 February 2020)). GC10-DET is a dataset of steel strip surface defects collected in a real-world manufacturing environment, with a total of 2312 gray-level images. The dataset comprises ten types of defects, including Punching (Pu), Welding line (Wl), Crescent gap (Cg), Water spot (Ws), Oil spot (Os), Silk spot (Ss), Inclusion (In), Rolled pit (Rp), Crease (Cr), and Waist folding (Wf). In terms of data scale and defect diversity, the GC10-DET dataset demonstrates superior performance compared with other datasets focusing on steel strip defects. The size, shape, and location of defects within this dataset exhibit variability, and there are no fixed patterns for defects of the same type. Furthermore, the number and types of defects present in each image are not constrained. Therefore, this dataset is closer to the benchmark of the actual scene. Sample images from the GC10-DET dataset are illustrated in Figure 1.

The performance of machine vision in defect detection is often hampered by external environmental factors, such as the shooting angle and distance, as well as lighting irregularities. These factors impact the quality of acquired images and diminish the overall detection accuracy. To mitigate the above challenges, we carried out a preprocessing step in which we eliminated some of the non-conforming defect images in the GC10-DET dataset. A total of 2136 gray-level steel strip images contained in the GC10-DET dataset were utilized for analysis. The dataset was split randomly into three subsets: training set, validation set, and test set in the ratio of 8:1:1 to ensure optimal training results. The dataset includes labeling information that details both the category and location of defects. To enhance the efficiency of the defect recognition model training, the 1709 raw images in the training set were resized to 640×640.

### 2.3. The Proposed LSD-YOLOv5

**Figure 2:**
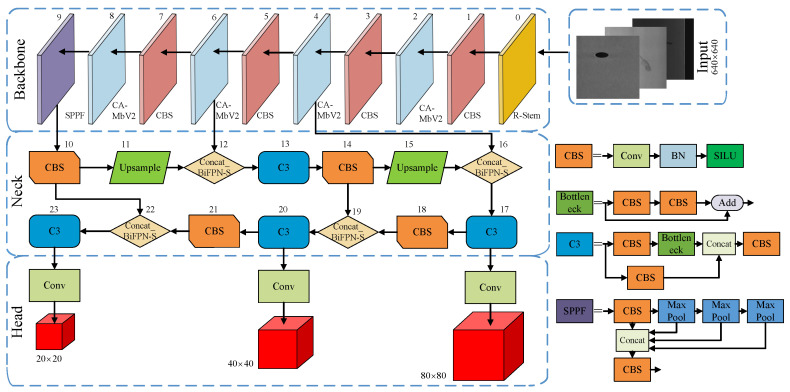
The structure of the LSD-YOLOv5 network.

The architecture of the LSD-YOLOv5 model is depicted in Figure 2. The LSD-YOLOv5 network comprises four primary modules: input, backbone, neck, and head. In the backbone network, the R-Stem module optimizes the capacity to capture low-level features, while the CBS module assists the CA-MbV2 module in feature extraction. The CA-MbV2 module minimizes the model parameters while boosting detection speed. In the neck network, the BiFPN-S module fuses the extracted feature information and generates three feature maps with different scales. Subsequently, the head detects and classifies objects by utilizing the generated feature maps. The post-processing phase of the model utilizes the Soft-DIoU-NMS algorithm for refined prediction boxes.

#### 2.3.1. The Backbone Network of LSD-YOLOv5

**Figure 3:**
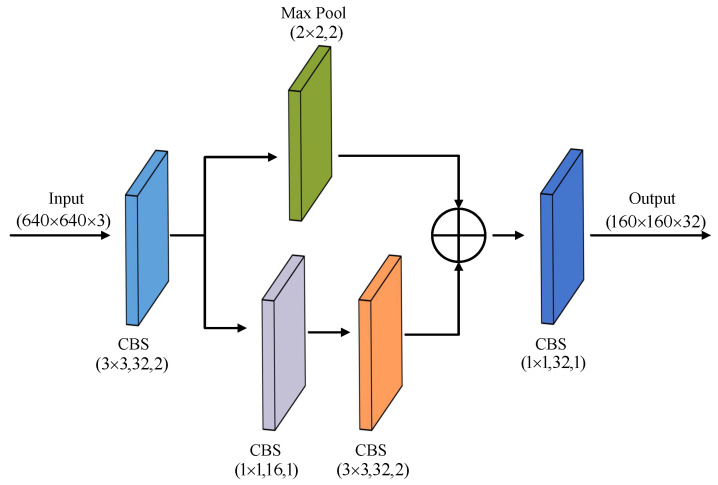
The structure of the R-Stem.

As depicted in Figure 3, we designed a new R-Stem module with reduced parameters and computation to replace the initial Conv structure in the backbone network. The R-Stem module can effectively enrich the features of steel strip defects and boost the overall generalization performance of the model [35], while the computational cost is almost unchanged. Based on the Stem module, we added a batch normalization operation and SiLU activation function after each Convolutional structure. The R-Stem module merges information from multiple channels for information fusion and splits the downsampling operation of the feature map into two branches. Specifically, one branch employs convolution while the other utilizes the maximum pooling operation. The resulting features from both branches are integrated and then passed through another convolution operation. The utilization of the R-Stem module accelerates model convergence, greatly mitigates the overfitting problem, and enhances the feature expression of the model.

**Figure 4:**
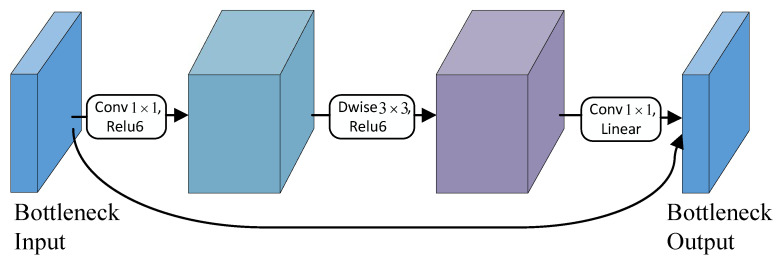
The bottleneck of MobileNetV2 structure.

The backbone network of YOLOv5 contains four C3 structures, resulting in a large number of model parameters and slow detection. Therefore, it is crucial to develop a lightweight feature extraction network. MobileNetV2 [36] is a convolutional neural network architecture designed specifically for resource-constrained mobile or embedded devices. It optimizes memory usage and execution speed, while maintaining high accuracy with minimal computational costs and parameters. This architecture performs equally well in resource-limited settings, considerably reducing the number of required operations and memory demands. The fundamental module of MobileNetV2 is the bottleneck depthwise separable convolution with residual connections, as depicted in Figure 4. A compressed representation of low dimensionality is first input and subsequently expanded to a higher dimension, which is then filtered using a lightweight depthwise convolution. Finally, the acquired high-dimensional features are remapped to the low-dimensional space through a linear convolution.

**Figure 5:**
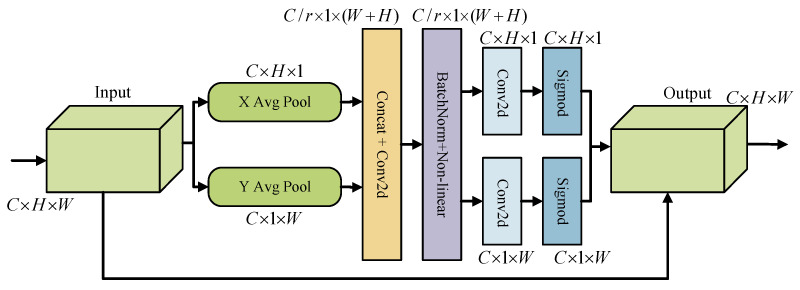
The structure of the Coordinate Attention mechanism.

Coordinate Attention (CA) is an efficient attention mechanism designed for lightweight networks, providing flexibility and low overhead. It can be easily integrated with various classical modules. The CA mechanism can capture cross-channel information, direction-aware, and position-aware information simultaneously, which greatly assists in accurately locating and identifying the target of interest. Therefore, we incorporated the CA mechanism into the MobileNetV2 bottleneck to better extract relevant information from the features. The CA mechanism encodes channel relationships and long-range dependencies by utilizing precise location information in two steps: coordinate information embedding and coordinate attention generation. The structure of the CA attention mechanism is shown in Figure 5. Given an input feature map *X*, we perform two separate one-dimensional pooling operations along the horizontal and vertical coordinates, denoted by (H,1) and (1,W), respectively, to encode each channel. The output of the *c*-th channel at the height *h* is shown in Equation (1). The output of the *c*-th channel at the width *w* is shown in Equation (2).

(1) $$Z_{c}^{h}\left(h\right)=\frac{1}{W}\sum_{0\leq i<W}x_{c}\left(h,i\right),$$

(2) $$Z_{c}^{w}\left(w\right)=\frac{1}{H}\sum_{0\leq j<H}x_{c}\left(j,w\right),$$

to generate coordinate attention, the feature maps aggregated through the aforementioned equations are combined, and then a shared 1×1 convolutional transform function $F_{1}$ is applied to the concatenated feature maps to obtain feature representation *f*, as shown in Equation (3).

(3) $$f=\delta (F_{1}\left(\left[z^{h},z^{w}\right]\right)),$$

where $f\in R^{C/r\times (w+h)}$ is the intermediate feature map that captures the spatial information along the horizontal and vertical coordinates, *r* denotes the scale of downsampling, and δ refers to the Sigmoid activation function. The feature map *f* is separated into two tensors along the spatial dimension and the feature maps $f^{h}$ and $f^{w}$ are converted into tensors with the same number of channels as the input *X* through two 1×1 convolutions, respectively, to obtain Equations (4) and (5).

(4) $$g^{h}=\sigma \left(F_{h}\left(f^{h}\right)\right),$$

(5) $$g^{w}=\sigma \left(F_{w}\left(f^{w}\right)\right),$$

where $g^{h}$ and $g^{w}$ are the attention weights in the height and width directions of the feature map. The final output of the CA module can be expressed by Equation (6):

(6) $$y_{c}\left(i,j\right)=x_{c}\left(i,j\right)\times g_{c}^{h}\left(i\right)\times g_{c}^{w}\left(j\right).$$

**Figure 6:**
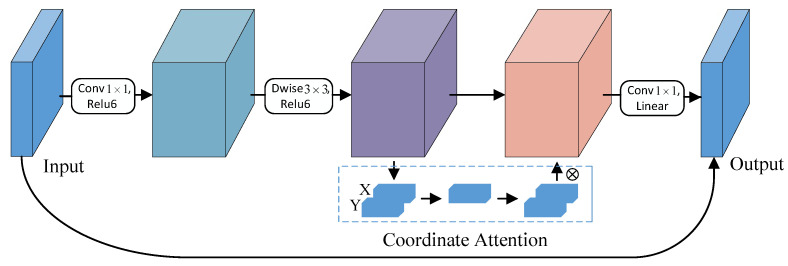
The CA-MbV2 module structure.

The structure of the CA-MbV2 module is shown in Figure 6. We proposed a highly efficient and lightweight network for feature extraction. Specifically, we replaced the first Conv structure in the backbone network with the R-Stem module, introduced the CA-MbV2 structure, and kept the SPPF module intact. The proposed network not only guarantees high detection accuracy, but also has a lightweight structure that satisfies real-time requirements for detecting surface defects on steel strips.

#### 2.3.2. The Feature Pyramid Network of LSD-YOLOv5

**Figure 7:**
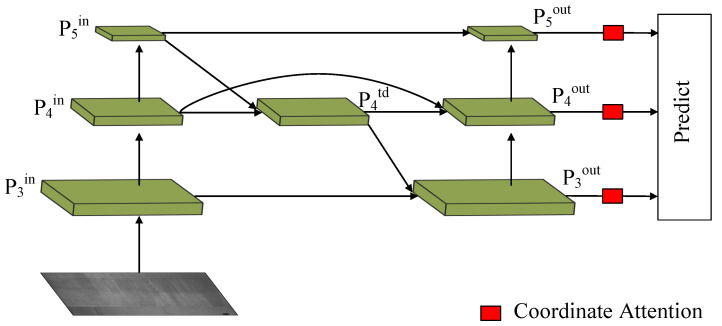
The structure of the BiFPN-S.

We found that the original model exhibited sub-optimal detection performance for small targets. The primary cause is the degradation of semantic details associated with small targets resulting from the multiple convolutions executed by the neural network. The Path Aggregation Network (PANet) achieves multi-scale feature fusion through upsampling and downsampling operations. However, PANet’s computational cost is significant, and its feature fusion approach simply merges different input features without explicit differentiation or weighting. Bi-directional Feature Pyramid Network (BiFPN) is a lightweight network architecture designed for application scenarios where computational or memory resources are limited [37]. It can effectively capture multi-scale feature information and improve the accuracy of detection. Therefore, to attain a better balance of surface defect information across different scales of steel strips, we designed an augmented feature fusion network based on BiFPN, called BiFPN-S. The structure of BiFPN-S is shown in Figure 7. We streamlined the quintuplet input strata of BiFPN-S to a trio of input strata, to integrate with the YOLOv5 framework. BiFPN shares one weight for all channels in each stratum of the feature map, which impedes the network’s ability to acquire multi-scale encoding. To address this, we introduced a separate CA attention mechanism in each prediction branch to differentiate the importance of various channels within the same feature stratum. We replaced PANet with BiFPN-S and combined Concat with BiFPN-S at layers 16, 20, 24, and 28 in the network architecture, which we referred to as Concat_BiFPN-S. The utilization of BiFPN-S results in the improved recognition ability of the model for multi-scale targets and enhanced recognition rate of small targets with surface defects on steel strips.

#### 2.3.3. The Non-Maximum Suppression of LSD-YOLOv5

During the inference stage of object detection, multiple prediction boxes can be generated for a single detection target, but only one accurate prediction box is required to be retained. YOLOv5 utilizes the traditional non-maximum suppression (NMS) approach to eliminate redundant prediction boxes. However, this algorithm exhibits suboptimal performance when overlapping or closely spaced defects exist on the steel strip surface. Due to the mutual occlusion of targets resulting in a significant overlapping region of prediction boxes, NMS may regard the occluded instance bounding boxes as redundant information and remove them from the final detection results. This results in diminished recall and missed detection, rendering it unsuitable for steel strip defect identification models that require precise detection.

**Figure 8:**
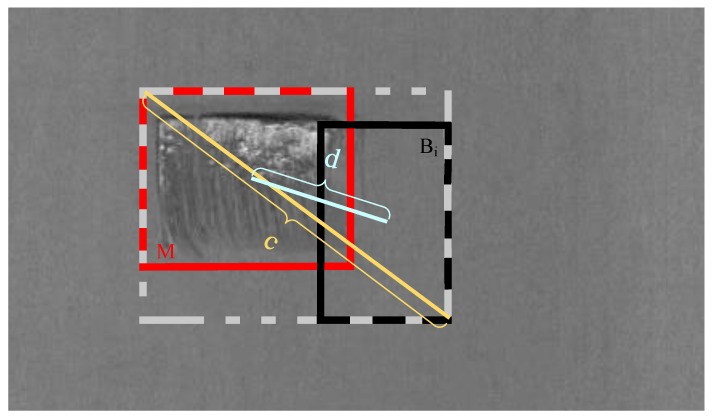
The calculation process of $R_{DIoU}$.

The DIoU-NMS algorithm [38] has alleviated the issue of suboptimal detection of occluded targets through the traditional NMS approach to some extent. However, closely spaced and mutually occluding defects still can result in missed detections. To address the issues, we improved the DIoU-NMS algorithm to obtain Soft-DIoU-NMS. The Soft-DIoU-NMS algorithm can compute the overlap between bounding boxes with greater precision, thereby more effectively retaining the key information of target detection results. This enhances the detection performance of occluded targets and diminishes the rate of missed detection. The Soft-DIoU-NMS algorithm is depicted as follows:

(7) $$S_{i}=\left\{\begin{array}{ccc} S_{i}, & & IoU-R_{DIoU}\left(M,B_{i}\right)<\xi \\ S_{i}\left(1-IoU\left(M,B_{i}\right)\right), & & IoU-R_{DIoU}\left(M,B_{i}\right)\geq \xi \end{array}\right.,$$

(8) $$R_{DIoU}=\frac{\rho^{2}\left(b,b^{gt}\right)}{c^{2}},$$

where $S_{i}$ represents the classification score, $R_{DIoU}$ denotes the penalty term as shown in Figure 8, *M* signifies the highest scoring prediction box, $B_{i}$ represents other prediction boxes, and ξ indicates the threshold value for NMS. Equation (8) defines $R_{DIoU}$, and $d=\rho (b,b^{gt})$ denotes the Euclidean distance between the central points of *M* and $B_{i}$. According to Equation (7), when the IoU value between the highest confidence prediction box and other prediction boxes is below the threshold value, the other prediction boxes remain unaltered; otherwise, the confidence of other prediction boxes decays until it falls below the threshold value, at which point the detection box is removed.

## 3. Experiments and Results

### 3.1. Experimental Environment and Parameter Setting

The experimental setup in this paper utilized an Intel Core i9-11900K @3.50GHz CPU, NVIDIA GeForce RTX3060 GPU, and Ubuntu 18.04 64-bit operating system. The experimental environment was built using the PyTorch framework, CUDNN 8.2, and CUDA 11.3. The model was trained using the Stochastic Gradient Descent (SGD) optimizer with a weight decay of 0.0005 and a momentum of 0.937. In this study, the initial learning rate was set to 0.001 and the minimum learning rate was set to 0.01 times the initial learning rate. The warm-up method is a prevalent learning rate optimization strategy commonly employed to enhance the stability of the deeper models. In the initial training phase, warm-up training was conducted for 5 epochs to update the learning rate of the model. After the warm-up phase, the cosine annealing algorithm was employed to dynamically regulate the learning rate variation. The batch size was set to 32 and the training process was conducted for a total of 200 epochs.

When working with a limited dataset, data augmentation techniques can be employed to increase the number of training samples as well as the diversity, thereby bolstering the robustness of the model. We used the Mosaic method to process images of surface damage on steel strips. By randomly cropping, scaling, and arranging 4 images into a single image and then inputting it into the network for training, we enriched the dataset and increased the number of small samples.

### 3.2. Evaluation Metrics

To evaluate the performance of the detection model, precision, recall, mean average precision (*mAP*), model parameters (Paramas), and detection speed (FPS) are commonly used as metrics. Precision is defined as the ratio of correctly predicted positive samples to the total number of predicted positive samples [39]. The recall is defined as the ratio of correctly predicted positive samples to the total number of actual positive samples [40]. The mathematical expressions for computing precision and recall are presented in Equations (9) and (10), respectively.

(9) $$Precision=\frac{TP}{TP+FP},$$

(10) $$Recall=\frac{TP}{TP+FN},$$

where *TP* represents the number of positive samples predicted as positive samples, *TN* is the number of negative samples predicted as negative samples, *FP* denotes the number of negative samples predicted as positive samples, and *FN* indicates the number of positive samples predicted as negative samples.

AP denotes the mean detection accuracy for each defect class, while *mAP* represents the mean detection accuracy across all defect classes. The equations for calculating *AP* and *mAP* are expressed in Equations (11) and (12), respectively. The increase in the number of model parameters can lead to a corresponding increase in both the size of the model file and memory usage. The FPS metric indicates the number of images that can be processed by the model per second and is used to evaluate whether the model meets the real-time detection requirements.

(11) $$AP=\int_{0}^{1}P\left(r\right)dr,$$

(12) $$mAP=\frac{1}{n}\sum_{i=1}^{n}AP_{i}.$$

### 3.3. Ablation Experiments and Analysis

To validate the effectiveness of each module incorporated into our proposed LSD-YOLOv5 model, we conducted ablation experiments using the GC10-DET dataset and employed YOLOv5s in version 6.1 of YOLOv5 as the baseline algorithm for comparison.

#### 3.3.1. Ablation Experiment of LSD-YOLOv5

In Section 2, we proposed the lightweight steel strip surface defect detection model LSD-YOLOv5. We conducted an ablation study by incrementally adding improvement modules. Table 1 presents the ablation results for each module. Bold indicates the best metric.

First, the original YOLOv5 backbone network was replaced by a lightweight MobileNetV2 structure. As shown in Table 1, the accuracy of the network model was 63.8%, representing a slight decrease of 1.7% compared with the original model. However, the model parameters and inference time were significantly reduced by 82.2% and 34.5%, respectively. This indicates that the network has a more lightweight architecture and faster detection capabilities, making it feasible for deployment on devices with limited memory and computation resources. Second, a new feature extraction network was constructed by incorporating the CA mechanism into the lightweight MobileNetV2 architecture. Compared with using the MobileNetV2 architecture as the backbone network, the mAP of the model was improved by 0.9%. This demonstrates that incorporating the CA attention mechanism makes the backbone network more focused on extracting useful information from the features. Subsequently, the R-Stem was implemented on the backbone network with a 1.2% improvement in mAP, while maintaining a minimal increase in model parameters and inference time. Moreover, the CA mechanism was placed in each prediction branch of the BiFPN and combined this new module with the Concat operation in the network. As presented in Table 1, the mAP of the model improved by 1.3%, demonstrating that BiFPN-S can better merge feature information for identifying steel strip surface damage. Finally, we utilized Soft-DIoU-NMS to refine the prediction boxes in the post-processing process of defect detection. This resulted in an improvement of 0.7% and 1.9% in mAP and Recall of the model, respectively, which reduced the missed detection rate of overlapping targets and improved the detection accuracy.

#### 3.3.2. Visualization Results on Ablation Experiment

**Figure 9:**
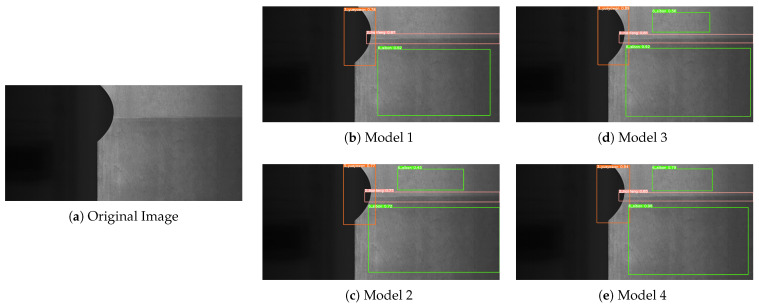
Detection results of inconspicuous defects. (**a**) Original image sample; (**b**) Model 1 detection results; (**c**) Model 2 detection results; (**d**) Model 3 detection results; (**e**) Model 4 detection results.

**Figure 10:**
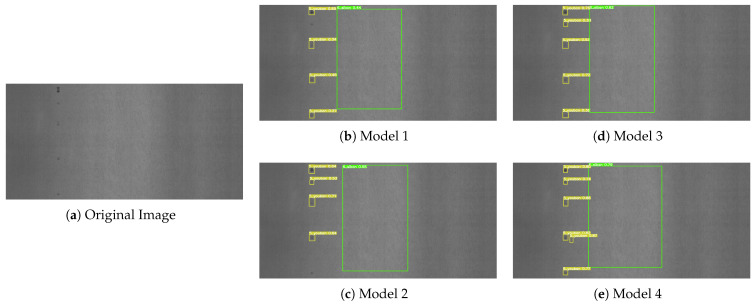
Detection results of small defects. (**a**) Original image sample; (**b**) Model 1 detection results; (**c**) Model 2 detection results; (**d**) Model 3 detection results; (**e**) Model 4 detection results.

**Figure 11:**
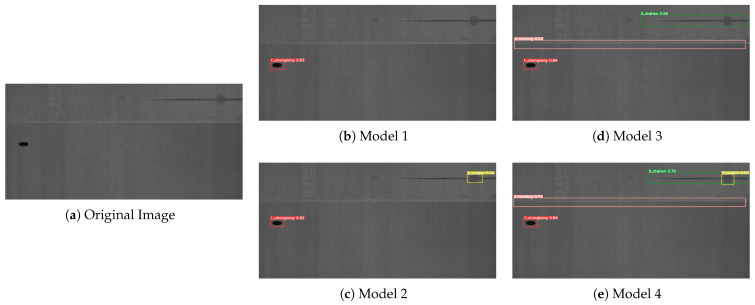
Detection results of overlapping defects. (**a**) Original image sample; (**b**) Model 1 detection results; (**c**) Model 2 detection results; (**d**) Model 3 detection results; (**e**) Model 4 detection results.

To more intuitively observe the experimental results presented in Table 1, we have provided some visual representations of the detection results in Figure 9, Figure 10 and Figure 11. Model 1 refers to the original YOLOv5s model, while Model 2 integrates the R-Stem and CA-MbV2 modules. Model 3 incorporates the BiFPN-S module based on Model 2, and Model 4 represents our proposed LSD-YOLOv5 model. We conducted a comparative analysis of the detection results with four models in three distinct scenarios.

From Model 1 to Model 4, there was a gradual increase in the number of detectable defects. And there were almost no missed inspections in the Model 4. As shown in Figure 9, compared with Model 1 detection, Model 2 was optimized by adding the R-Stem and CA-MbV2 modules to improve the detection accuracy for relatively faint, inconspicuous defects. As indicated in Figure 10, we performed a series of experiments to evaluate the performance of the four models in the scenario of small damage detection. Model 3 exhibited a substantial enhancement in the detection performance compared to the two preceding models. As depicted in Figure 10d, it accurately identified relatively small defects in the images, effectively improving the detection performance for multi-scale targets. However, Model 3 was susceptible to confusion when there were overlapping or closely located defects as shown in Figure 11. By using the Soft-DIoU-NMS algorithm in Model 4, as indicated in Figure 11e, the model could identify and accurately locate targets in proximity or overlap, thereby reducing the rate of missed detections.

### 3.4. Comparative Experiments and Analysis

**Figure 12:**
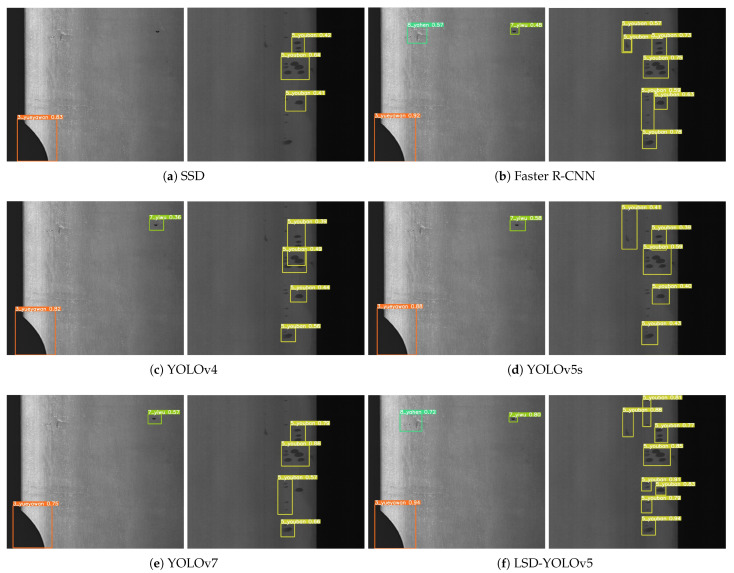
Comparison of visualization results using different detection algorithms.

To further demonstrate the reliability and validity of our proposed model, we compared LSD-YOLOv5 with popular algorithms in object detection including SSD [41], Faster R-CNN [42], YOLOv4, YOLOv5s, and YOLOv7. The experimental results are presented in Figure 12 and Table 2. In industrial settings, the task of detecting surface defects on steel strips must consider not only accuracy but also the optimization of detection efficiency and model weight. Therefore, in this section of the discussion, Precision, mAP, Recall, model parameters (Paramas), floating-point operations (Flops), and frames per second (FPS) are used as comprehensive evaluation metrics for the detection model.

Figure 12 shows the visualization of the recognition effects, where the labels on the anchor boxes indicate the confidence scores of the model detection. The visualization shows that our proposed model has a high confidence score in the damaged area and the recognition result is accurate. Furthermore, the model exhibits excellent detection performance for inconspicuous defects, such as “Rolled pit” (marked as “8_yahen”), and small-scale defects, such as “Oil spot” (marked as “5_youban”). As shown in Table 2, the Faster R-CNN algorithm had the highest mAP and Recall of 68.2% and 67.8%. However, its model parameters and computational demands of 63.57 M and 263.5 G were considerably larger than other detection models. Additionally, the Faster R-CNN model had an FPS of 12.6, which falls short of real-time detection requirements. The mAP achieved by YOLOv7 was inferior to that of YOLOv5s, and the model entailed higher parameters and computational demands, rendering it unsuitable for deployment in application scenarios with limited resources, such as mobile devices, etc. The LSD-YOLOv5 model proposed in this paper exhibited strong capabilities in terms of detection accuracy and speed. Specifically, the mAP of our proposed model was 67.9%, representing an improvement of 2.4% over the YOLOv5s model. Moreover, our proposed model achieved an FPS of 90.1, representing a 28.7% increase compared to the previous 70 FPS. This enhancement has resulted in increased efficiency and suitability for real-time detection in steel strip production environments. Finally, LSD-YOLOv5 had significant advantages in terms of model parameters and computational complexity, with only 2.71 M parameters and 9.1 G computational requirements. Compared with the original model, the model parameters and computation were reduced by 61.5% and 42.8%, highlighting the effectiveness of our approach in achieving a lightweight network architecture. In summary, LSD-YOLOv5 delivers enhanced accuracy in detecting defects, with a lightweight model structure designed to meet the real-time demands of industrial detection.

### 3.5. Performance Analysis

**Figure 13:**
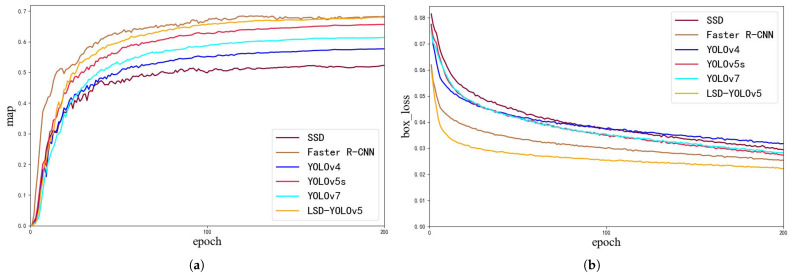

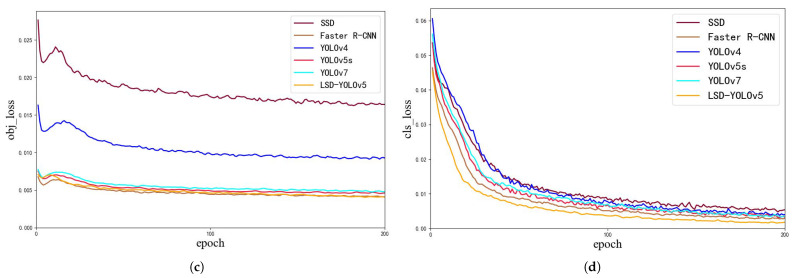
Comparison of evaluation indexes under different models. (**a**) mAP curve. (**b**) Bounding box loss curve. (**c**) Confidence loss curve. (**d**) Classification loss curve.

Figure 13 illustrates the comparison of evaluation indexes for different models. In Figure 13a, the mAP of our proposed model LSD-YOLOv5 was 67.9%, which was 2.4% higher than the original model. The convergence speed and stability of the model can provide a better assessment of the model’s performance. As shown in Figure 13, the loss curve had a larger drop at the beginning of the model training, indicating that the initial hyperparameter setting was reasonable. All other parameters held constant, the improved model had a steeper convergence curve in the first period. After a period of training, the loss curve gradually leveled off and the model converged. It can be seen that the improved YOLOv5s model has a more stable convergence state and higher convergence efficiency than the original model. Finally, as depicted in Figure 13b, the bounding box loss curve of the LSD-YOLOv5 model converged significantly faster than that of the original model, which can be attributed to the effectiveness of the CA attention mechanism in mitigating the effects of extraneous features and enhancing the extraction of relevant information.

## 4. Conclusions

In this paper, we have proposed an optimal LSD-YOLOv5 model for recognizing surface damage on steel strips in real-world scenarios, which is characterized by fewer parameters and lower computational requirements. The model strikes an ideal balance between detection accuracy and speed, while maintaining a lightweight structure. First, to improve the feature extraction capability of shallow networks, the R-Stem module was proposed to replace the first Conv structure in the backbone network. And, we designed a new feature extraction network called CA-MbV2, which integrates the Coordinate Attention mechanism into the bottleneck structure of MobileNetV2. CA-MbV2 has significantly reduced the model parameters and improved the detection speed. Second, we introduced BiFPN-S into the neck layer and placed a lightweight attention module in each prediction branch, enhancing the model’s ability to modulate various scales of damage. Finally, Soft-DIoU-NMS was utilized as a prediction frame screening algorithm to minimize the number of missed detections of overlapping targets.

The effectiveness of the proposed method has been demonstrated by conducting extensive evaluations and ablation studies on the GC10-DET dataset. In comparison with the original YOLOv5s model, the proposed LSD-YOLOv5 model has achieved a reduction of 61.5% in parameters, a decrease of 42.8% in computation, and a 2.4% improvement in accuracy, making it more suitable for meeting the lightweight requirements of steel strip surface defect detection. This study has the potential to provide insights into lightweight and real-time methods for detecting metal surface defects in industrial settings, and may establish a basis for the implementation of industrial automation. In the future, we will consider exploring the adoption of more efficient data augmentation techniques, such as Generative Adversarial Networks (GAN), to enhance the recognition and generalization capabilities of our models.

## Figures and Tables

**Table 1:** Ablation study on LSD-YOLOv5.

Model	Precision (%)	mAP (%)	Recall (%)	Params (M)	Inference Time (ms)
YOLOv5s(baseline)	67.6	65.5	62.3	7.04	14.2
+MobileNetV2	64.1	63.8	61.4	**1.25**	**9.3**
+CA-MbV2	66.3	64.7	62.6	1.83	9.9
+R-Stem+CA-MbV2	67.7	65.9	63.5	1.98	10.1
+R-Stem+CA-MbV2+BiFPN-S	68.9	67.2	64.9	2.71	10.7
LSD-YOLOv5	**69.8**	**67.9**	**66.8**	2.71	11.1

**Table 2:** Comparison of detection results using different detection algorithms.

Model	Precision (%)	mAP (%)	Recall (%)	Params (M)	Flops (G)	FPS
Faster R-CNN	68.6	**68.2**	**67.8**	63.57	263.5	12.6
SSD	53.3	51.6	52.1	27.32	39.2	54.7
YOLOv4	60.2	57.5	56.3	52.36	121.3	41.5
YOLOv5s	67.6	65.5	62.3	7.04	15.9	70
YOLOv7	62.0	61.3	60.6	37.21	103.6	56.3
LSD-YOLOv5	**69.8**	67.9	66.8	**2.71**	**9.1**	**90.1**

## Data Availability

Publicly available datasets were analyzed in this study. These data can be found here: https://github.com/lvxiaoming2019/GC10-DET-Metallic-Surface-Defect-Datasets (accessed on 24 February 2020).
